# Human Neuronal Cell Lines as An In Vitro Toxicological Tool for the Evaluation of Novel Psychoactive Substances

**DOI:** 10.3390/ijms22136785

**Published:** 2021-06-24

**Authors:** Valeria Sogos, Paola Caria, Clara Porcedda, Rafaela Mostallino, Franca Piras, Cristina Miliano, Maria Antonietta De Luca, M. Paola Castelli

**Affiliations:** 1 Department of Biomedical Sciences, University of Cagliari, 09042 Monserrato, Italy; sogos@unica.it (V.S.); paola.caria@unica.it (P.C.); porcedda.clara@gmail.com (C.P.); rafaela.mostallino@unica.it (R.M.); fpiras@unica.it (F.P.); deluca@unica.it (M.A.D.L.); 2 School of Neuroscience, Virginia Polytechnic Institute and State University, Blacksburg, VA 24060, USA; cristinamiliano@hotmail.it; 3 Guy Everett Laboratory, University of Cagliari, 09042 Monserrato, Italy; 4 Center of Excellence “Neurobiology of Addiction”, University of Cagliari, 09042 Monserrato, Italy

**Keywords:** cytotoxicity, oxidative stress, apoptosis, Bax and Bcl2 expression, dopaminergic cells, cathinone, phenethylamine, fentanyl

## Abstract

Novel psychoactive substances (NPS) are synthetic substances belonging to diverse groups, designed to mimic the effects of scheduled drugs, resulting in altered toxicity and potency. Up to now, information available on the pharmacology and toxicology of these new substances is very limited, posing a considerable challenge for prevention and treatment. The present in vitro study investigated the possible mechanisms of toxicity of two emerging NPS (i) 4′-methyl-alpha-pyrrolidinoexanophenone (3,4-MDPHP), a synthetic cathinone, and (ii) 2-chloro-4,5-methylenedioxymethamphetamine (2-Cl-4,5-MDMA), a phenethylamine. In addition, to apply our model to the class of synthetic opioids, we evaluated the toxicity of fentanyl, as a reference compound for this group of frequently abused substances. To this aim, the in vitro toxic effects of these three compounds were evaluated in dopaminergic-differentiated SH-SY5Y cells. Following 24 h of exposure, all compounds induced a loss of viability, and oxidative stress in a concentration-dependent manner. 2-Cl-4,5-MDMA activates apoptotic processes, while 3,4-MDPHP elicits cell death by necrosis. Fentanyl triggers cell death through both mechanisms. Increased expression levels of pro-apoptotic Bax and caspase 3 activity were observed following 2-Cl-4,5-MDMA and fentanyl, but not 3,4-MDPHP exposure, confirming the different modes of cell death.

## 1. Introduction

In the early 2000s, the United Nations Office on Drugs and Crime (UNODC) defined designer drugs as “novel chemical substances with psychoactive properties,” synthetized based on chemical structures of well-known drugs of abuse, and clandestinely manufactured with the intention to mimic the effects of controlled substance and circumvent regulation [1]. Later, UNODC coined the new term “new psychoactive substances” (NPS) to designate “*substances of abuse, either in a pure form or a preparation, that are not controlled by the 1961 Single Convention on Narcotic Drugs or the 1971 Convention on Psychotropic Substances, but which may pose a public health threat*” (UNODC, www.unodc.org). NPS are a broad range of drugs belonging to different chemical classes able to elicit stimulant, entactogen, hallucinogen, depressant, and cannabis-like effects with higher potency compared to the original compounds [2,3]. Around 830 NPS were monitored by the EMCDDA in 2020, and 46 of them were first identified in 2020 [4]. Roughly, 500 NPS are found on the national markets of Member States each year [5] and, over time, the trends and patterns of use change, posing a great challenge for their detection, notification, and legal banning. The EMCDDA harmonizes the action of each national agency against NPS (i.e., emergences, seizures, and poisonings data collection and analysis) and shares information with the UNODC for an exhaustive analysis [6].

Two main factors strongly support the worldwide NPS market: their ambiguous legal status and their availability on both the surface and deep web, as well as social networks and smartphone apps [7]. 

In the global framework, Europe, supported by the production in other regions such as Latin America, West Asia, and North Africa, acts as a pivotal market and transit for drugs and precursors. Unfortunately, the lack of international consensus on the drug policy of the NPS prevents a quick identification and the risk assessment needed for facing this social problem [8]. 

Among different NPS classes, synthetic cathinones and phenethylamines are currently the second and third largest group of NPS after synthetic cannabinoids, with 138 and 99 different derivatives molecules, respectively [4,9]. Synthetic cathinones, sold as crystalline powder, tablets, and capsules and generally labelled as “bath salts” or “plant fertilizers,” include heterogeneous cocaine- and amphetamine-like compounds derived from cathinone, a monoamine alkaloid found in the Khat plant (*Catha edulis*) [10]. Cathinones cause a substantial efflux of monoamines from presynaptic terminals by either blocking presynaptic reuptake transporters for monoamines (dopamine, DAT; norepinephrine, NET; serotonin, SERT), or by acting as a substrate for these transporters and the vesicular monoamine transporter 2 (VMAT2) [11,12,13,14]. Exactly like almost all drugs of abuse, their rewarding effects are related to their ability to increase extracellular DA levels in the ventral striatum [15,16]. Following the ingestion of synthetic cathinones, people have experienced serious central and peripheral effects [17], such as agitation, tachycardia, hypertension, hyponatremia, chest pain, palpitations, hallucinations, and psychosis often associated with aggressive behaviors [9,18,19,20,21]; it should be noted that these effects may differ substantially in male and female users [22]. Phenethylamines, also known as “party pills,” refer to a class of substances that includes: (i) older drugs, such as amphetamine and MDMA, controlled under the 1971 Convention [23]; and (ii) novel phenethylamines, such as 4-fluoroamphetamine (4-FA), benzofurans (5-APB and 6-APB), 2C series compounds (e.g., 2C-B and 2C-E), and NBOMes (25B-NBOMe, 25C-NBOMe, and 25I-NBOMe) [24]. Based on their mechanism of action, these compounds have been distinguished in non-hallucinogenic (amphetamine, 4-FA, MDMA, 5-APB, and 6-APB) and hallucinogenic stimulant phenethylamines (2C-B and NBOMe series). The group of non-hallucinogenic ones is thought to act through the inhibition and/or the reversal of plasma membrane monoamine reuptake transporters (DAT, NET, and SERT), mimicking the effect of traditional psychostimulants (i.e., cocaine, amphetamine, and MDMA) [25,26], while the hallucinogenic ones act as agonists at 5-HT2A/B/C receptors [24,27]. Accordingly, exposure to phenethylamines induces stimulatory and/or entactogenic and psychedelic effects. Reported adverse effects of psychedelic phenethylamines, including agitation, mydriasis, confusion, hallucinations, aggression, hyperthermia, hypertension, and tachycardia, are shared with psychedelics of other chemical classes. Yet, seizures, cerebral edema, coma, long-lasting severe neurological impairment, serotonin syndrome, prolonged respiratory failure, multiorgan failure, and metabolic acidosis have also been described in several users [28,29,30,31,32,33,34,35,36]. 

Preclinical in vitro studies demonstrated that cathinones (i.e., mephedrone, methylone, MDPV, α-PNP, naphyrone, or 3-fluoromethcathione) are able to induce oxidative stress and mitochondrial dysfunction, reduce cell proliferation, and promote autophagy, necrosis, and/or apoptosis as measured by chromatin condensation in the neuronal cell lines HT22, SK-N-SH, and TGW [37,38,39,40,41,42]. Moreover, the NBOMes compounds have been reported to induce cardiotoxicity and neurotoxicity in the neuronal cell lines SH-SY5Y, PC12, and SN4741. 25D-NBOMe and 25C-NBOMe reduce the viability of H9c2 cells (cardiomyocytes), while 25C-NBOMe induces neurotoxicity through inhibition of the Akt pathway and the MAP/ERK cascade [43]. Recently, Cocchi et al. (2020) reported that the phenethylamines 2C-H, 2C-I, 2C-B, and 25B-NBOMe are genotoxic in the human lymphoblastoid TK6 cells [44]. Additionally, the effects of cathinones and phenethylamines have been studied in vivo by using animal models [24,45,46,47,48,49]. Moreover, high-resolution methods have been developed to detect these classes of NPS in different kinds of biological specimens (e.g., urine, hair, and plasma) [50,51]. 

Despite the advancement in this field of study, the number of NPS in the drug market is rising continuously, as well as the cases of intoxication. On the other hand, the in vivo preclinical models mentioned here are very expensive, and time- and resource-consuming, and their outcome is slow compared to the appearance of the NPS in the drug market. For these reasons, a valid in vitro toxicological method in human cells would represent an effective strategy of quick screening that has not been reported yet. Human SH-SY5Y cells are frequently used in vitro to model both undifferentiated and differentiated neuronal cells [52]. These cells express dopaminergic markers, such as DAT and tyrosine-hydroxylase (TH), and, if stimulated with several agents, show a mature neuron-like phenotype characterized by fusiform shape, extensive outgrowth of neurites, and increased expressions of TH and neuronal markers (i.e., neuron-specific enolase, synaptophysin, and synaptic-associated protein-97) [53,54,55,56]. This cell line is thus a useful research tool to reveal the underlying toxicity mechanisms of several drugs of abuse that have neurotoxic effects mediated by the dopaminergic system. 

**Figure 1:**
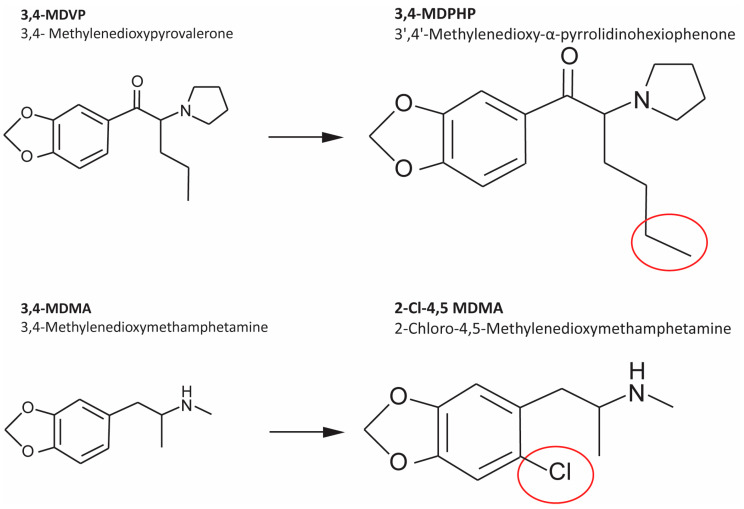
Chemical structures of 3,4-MDVP, 3,4-MDPHP, 3,4-MDMA, and 2-Cl-4,5-MDMA.

3,4-MDPHP and 2-Cl-4,5-MDMA are considered emerging NPS of interest. 3,4-MDPHP is an analog of 3,4-MDPV that differs only by the addition of a single carbon to the alkyl chain (Figure 1). Its synthesis was initially reported in the 1960s [57,58]. 3,4-MDPHP was notified to the EMCDDA by Sweden in 2014. 3,4-MDPHP is a potent monoamine transporters inhibitor [59,60,61]. Acute intoxication is characterized by tachycardia, hypertension, various cardiovascular effects, as well as agitation, hallucinations, and paranoia [59]. A case of fetal death associated with the use of 3,4-MDPHP has been reported [62]. Gas and liquid chromatography–mass spectrometry are mainly used for the identification of synthetic cathinones, including 3,4-MDPHP, in seized and forensic case samples [63], as well as biological specimens (blood, urine, hair, oral fluid, and tissue) [64]. 2-Cl-4,5-MDMA, also called 6-Cl-MDMA, is an amphetamine derivative of MDMA with high toxicity by ingestion, inhalation, and contact [65]. This phenethylamine was first identified, by gas chromatography coupled with mass spectrometry (GC-MS) and NMR, in a batch of seized MDMA (“ecstasy”) tablets [66]. It was later classified as a psychostimulant and euphoric. The structure of 2-Cl-4,5-MDMA was clarified in a study in 2005 where the goal was to identify an unknown compound that had been detected by thin-layer chromatography (TLC) in the urine of a drug addict; later, the compound was isolated by TLC and then analyzed by GC-MS [65]. Based on the data provided, the compound differs from MDMA only for the addition of a chlorine atom (see Figure 1); for this reason, it has been considered a possible synthetic impurity [67]. The legal status of 3,4-MDPHP and 2-Cl-4,5-MDMA is still ambiguous, but their main congeners are scheduled in several countries (e.g., UK, USA, Italy, Germany, China, and Canada).

Assessing the potential neurotoxicity induced by NPS, such as 3,4-MDPHP and 2-Cl-4,5-MDMA, is of paramount importance to help Regulatory Agencies in the early identification of dangerous substances appearing in the drug market. Therefore, in the present study, we used human dopaminergic neurons derived by retinoic acid differentiation of the SH-SY5Y cell line to evaluate in vitro the neurotoxic effects of the cathinone 3,4-MDPHP, and the phenethylamine 2-Cl-4,5-MDMA (Figure 1) by assessing cell viability, oxidative stress, induction of apoptosis/necrosis, and mitochondrial alterations. 

Moreover, we applied our in vitro model to the class of synthetic opioids, as well by evaluating the toxicity of fentanyl, one of the most popular and frequently abused among this class [68]. Providing evidence that three classes of NPS induce in vitro neurotoxicity, the present study might highlight the utility of in vitro testing as a quick and efficient test to screen new compounds.

## 2. Results

### 2.1. 3,4-MDPHP, 2-Cl-4,5-MDMA, and Fentanyl Reduced Cell Viability on SH-SY5Y-Differentiated Cells

The neurotoxic potential of 3,4 MDPHP, 2-Cl-4,5-MDMA, and fentanyl was evaluated by using two well-validated assays such as MTT [69] and LDH in the cell model of dopaminergic-differentiated SH-SY5Y [70]. SH-SY5Y cells were treated with increasing concentrations (15–1000 μM) of 3,4-MDPHP, 2-Cl-4,5-MDMA, and fentanyl for 24 h. All tested compounds induced loss of cell viability in a concentration-dependent manner.

**Figure 2:**
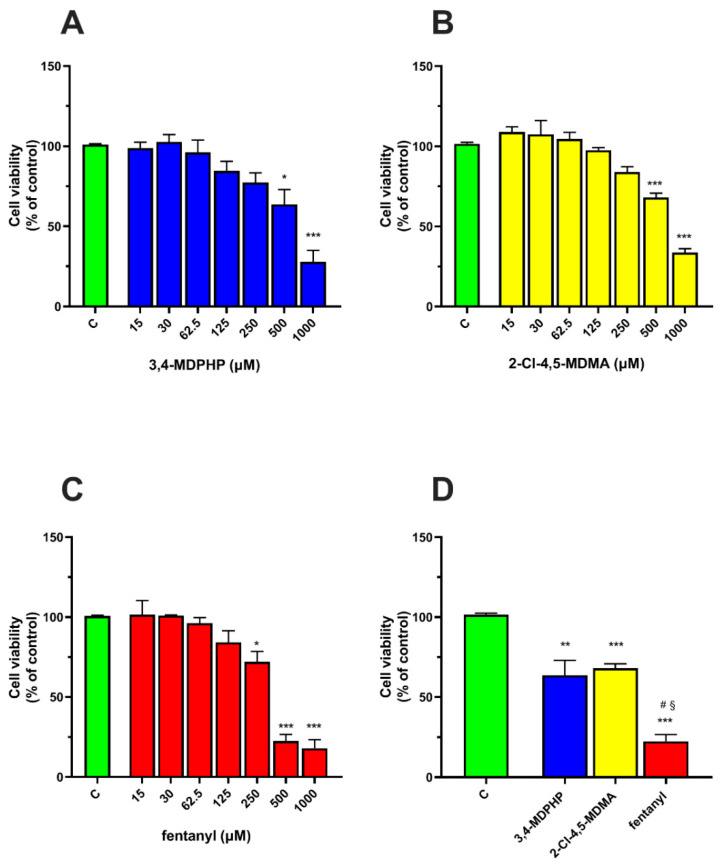
3,4-MDPHP, 2-Cl-4,5-MDMA, and fentanyl induced cell viability loss in a concentration-dependent manner. Dopaminergic-differentiated SH-SY5Y cells were exposed for 24 h to the different concentrations of 3,4-MDPHP (**A**), 2-Cl-4,5-MDMA (**B**), and fentanyl (**C**), or to 500 µM of the indicated substance (**D**), and then their viability was measured using the MTT assay. Cell viability was expressed as a percentage of cell viability relative to the control (untreated cells). Data are representative of three independent assays with six replicates for each concentration of the substance used and are presented as mean ± SEM. One-way ANOVA followed by Tukey’s post-hoc. * *p* < 0.05, ** *p <* 0.01, *** *p <* 0.001 compared to respective vehicle-treated control; § *p <* 0.001 with respect to 2-Cl-4,5-MDMA and # *p <* 0.01 compared to 3,4-MDPHP.

**Figure 3:**
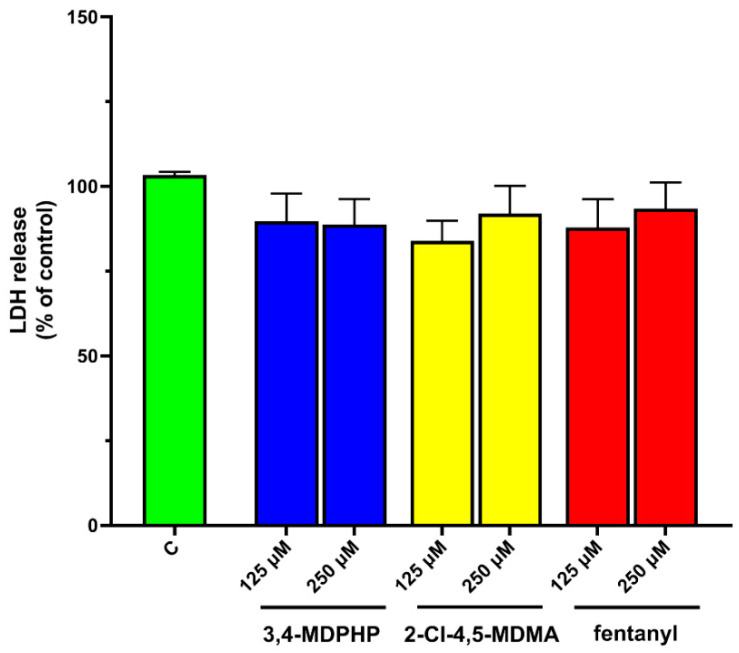
3,4-MDPHP, 2-Cl-4,5-MDMA, and fentanyl failed to modify membrane integrity evaluated by the LDH release assay. No effect was observed in the LDH release assay, indicating cell membrane integrity after exposure for 24 h with two concentrations (125 and 250 µM) of the indicated drugs. Data expressed as mean ± SEM are representative of three independent assays with three replicates for each concentration of the compounds.

As shown in Figure 2A,B, 3,4-MDPHP and 2-Cl-4,5-MDMA caused a significant reduction in cell viability at the highest concentrations of 500 and 1000 μM. With respect to control values, both compounds at a concentration of 500 μM induced significant decreases of 36% (*p* < 0.05) and 32% (*p <* 0.001) for 3,4-MDPHP and 2-Cl-4,5-MDMA, respectively. The maximum effect on SH-SY5Y viability (about 72% and 76% reduction for 3,4 MDPHP and 2-Cl-4,5-MDMA, respectively) was observed at the highest concentration tested (i.e., 1000 μM, *p <* 0.001). 

Finally, fentanyl induced a significant decrease in cell viability from 250 to 1000 μM; a 28% reduction with respect to the control was observed at 250 μM (*p <* 0.05), whereas the maximum effect was detected at concentrations of 500 and 1000 μM (78% and 82% decrease with respect to the control, respectively; *p <* 0.001) (Figure 2C). In addition, one-way ANOVA analysis revealed that at the dose of 500 μM, fentanyl was the most potent compound, while no difference was observed between 3,4-MDPHP and 2-Cl-4,5-MDMA. Tukey’s post-hoc test showed that fentanyl caused a significant decrease in cell viability with respect to the control (*p <* 0.001), 3,4-MDPHP (*p <* 0.01), and 2Cl-4,5-MDMA (*p <* 0.001) (Figure 2D).

We next examined whether exposure to our compounds at the concentration of 125 or 250 μM induced cytotoxic cell damage by assessing LDH leakage. After 24 h of treatment with fentanyl, 3,4-MDPHP, or 2-Cl-4,5-MDMA, no difference in the LDH release was observed between treated and control cells (Figure 3), indicating that the reduction in cell viability was not caused by loss of cell membrane integrity.

### 2.2. Mechanisms of 3,4-MDPHP, 2-Cl-4,5-MDMA, and Fentanyl Toxicity in SH-SY5Y Cells 

Based on MTT results, two concentrations (125 and 250 μM) of 2-Cl-4,5-MDMA, 3,4-MDPHP, and fentanyl were used to explore the underlying mechanisms of cell death, such as the involvement of ROS production and/or of apoptotic/necrotic processes. For this purpose, the assessment of reactive oxygen species levels and of apoptotic/necrotic cells was performed.

#### 2.2.1. 3,4-MDPHP, 2-Cl-4,5-MDMA, and Fentanyl Increased ROS Levels on SH-SY5Y-Differentiated Cells

To address the role of oxidative stress in the neurotoxic effect of our compounds, we measured the intracellular levels of ROS using DCFDA-DA as a fluorescent probe. Levels of intracellular ROS were monitored by utilizing the DCFH-DA probe, which diffuses through the cell membrane and turns into a green, fluorescent dye in the presence of intracellular ROS.

**Figure 4:**
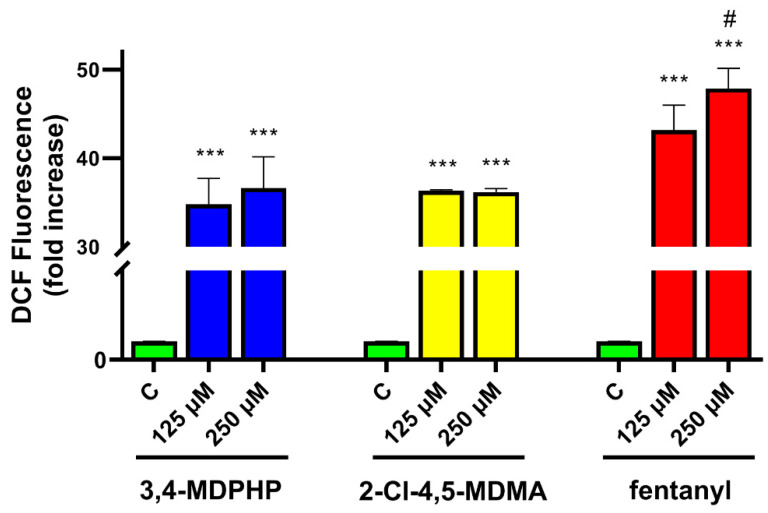
3,4-MDPHP, 2-Cl-4,5-MDMA, and fentanyl increased ROS production in dopaminergic-differentiated SH-SY5Y cells. Dopaminergic-differentiated SH-SY5Y cells were treated for 24 h with compounds (125 and 250 μM) or culture medium containing 1% of DMSO as a negative control. Fluorescence data from DCFH-DA assay were normalized to negative controls (no drug exposure, set to 1), expressed as mean ± SEM, and were from three independent experiments with three replicates for each concentration of the compounds. Two-way ANOVA followed by Tukey’s post-hoc. *** *p* < 0.001 compared to their respective controls and # *p <* 0.05 vs. 3,4-MDPHP and 2-Cl-4,5-MDMA.

Twenty-four hours after drug treatments, two-way ANOVA (dose x treatment) of ROS levels showed the main effect of treatment (F_(2,18)_ = 383.80, *p <* 0.0001), dose (F_(2,18)_ = 10.58, *p* = 0.0009), and treatment x dose interaction (F_(4,18)_ = 3.03, *p* = 0.0447). Tukey’s post-hoc tests revealed that treatment with 3,4-MDPHP, 2-Cl-4,5-MDMA, or fentanyl (125 or 250 μM) induced significant increases in ROS levels with respect to their respective control (*p <* 0.0001) (Figure 4). Additionally, as shown in Figure 4, the highest dose of fentanyl induced a significantly higher ROS production with respect to 250 μM of 3,4-MDPHP, or 2-Cl-4,5-MDMA (*p <* 0.05; Tukey’s post-hoc test).

#### 2.2.2. 2-Cl-4,5-MDMA and Fentanyl, But Not 3,4-MDPHP, Induced Apoptosis in Differentiated SH-SY5Y Cells 

To determine whether 3,4-MDPHP, 2-Cl-4,5-MDMA, or fentanyl induce cell death through apoptosis or necrosis, we performed a double staining with fluorescent Annexin V-FITC and PI in differentiated SH-SY5Y. We assessed the externalization of phosphatidylserine by annexin V binding, and the permeability to propidium iodide (PI), as markers of apoptosis and necrosis, respectively. Analysis of apoptosis by two-way ANOVA (dose x treatment) revealed the main effect of treatment (F_(2,18)_ = 134.3, *p <* 0.0001), dose (F_(2,18)_ = 94.31, *p <* 0.0001), and treatment x dose interaction (F_(4,18)_ = 35.28, *p <* 0.0001).

**Figure 5:**
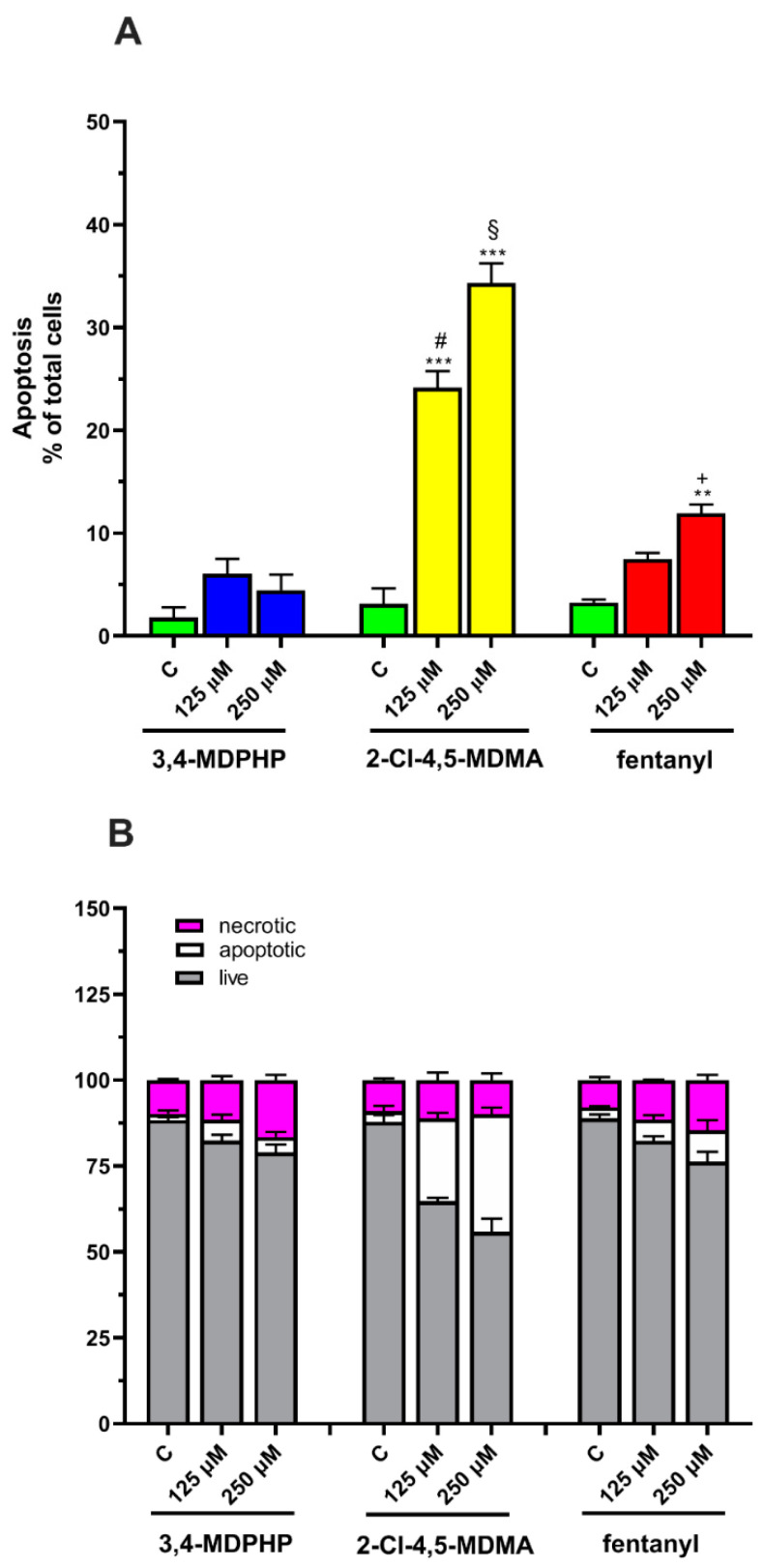
Dopaminergic-differentiated SH-SY5Y cell apoptosis is induced by 2-Cl-4,5-MDMA and fentanyl but not by 3,4-MDPHP. Percentage of apoptosis (**A**) and of live, apoptotic, and necrotic dopaminergic-differentiated SH-SY5Y cells (**B**) after 24 h of treatment with 3,4-MDPHP, 2-Cl-4,5-MDMA, or fentanyl determined by flow cytometry analysis. Data, expressed as % of total cells, represent mean ± SEM from three independent experiments with three replicates for each concentration of the compounds. Two-way ANOVA followed by Tukey’s post-hoc. ** *p* < 0.01 and *** *p* < 0.001 compared to their respective controls; # *p <* 0.001 compared to 125 μM of 3,4-MDPHP and fentanyl; ^§^ *p <* 0.001 compared to 250 μM of 3,4-MDPHP and fentanyl; + *p <* 0.05 compared to 250 μM of 3,4-MDPHP.

Tukey’s post-hoc test showed that 2-Cl-4,5-MDMA treatment induced a significant increase in apoptotic cells (*p <* 0.001) at both concentrations (125 and 250 μM) with respect to its own control, while fentanyl induced an increase in apoptotic cells only at the highest concentration tested (*p <* 0.01 vs. its own control) (Figure 5A,B). On the contrary, at both concentrations, 3,4-MDPHP failed to induce a significant increase in apoptosis. Moreover, as shown in Figure 5A,B, 2-Cl-4,5-MDMA (125 and 250 μM) treatment induced a significant increase in apoptotic cells as compared to the same doses of fentanyl (*p <* 0.001) and 3,4-MDPHP (*p <* 0.001). Finally, fentanyl at the highest concentration tested caused a significant increase (*p <* 0.05) in apoptotic cells compared to 250 μM of 3,4-MDPHP (Figure 5A,B). As shown in Table 1, the distribution of apoptotic cells upon treatment with 2-Cl-4,5-MDMA and fentanyl belonged predominantly to those with early apoptosis features. 

**Figure 6:**
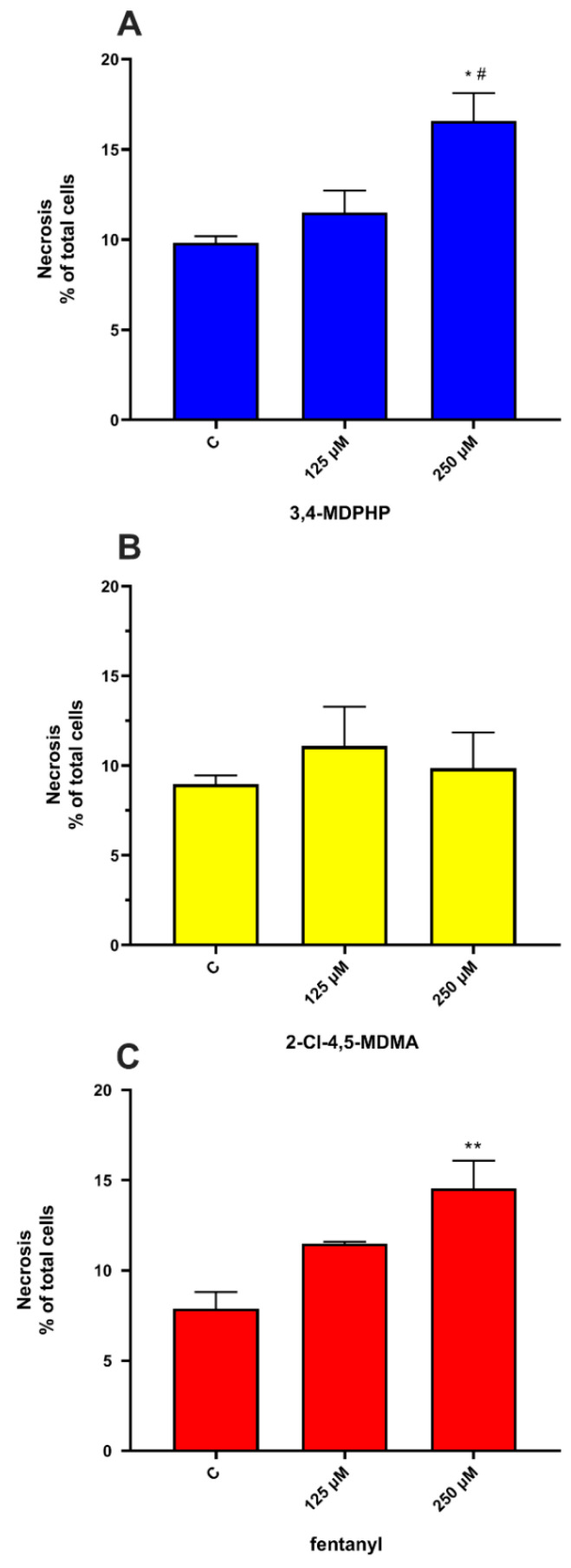
Dopaminergic-differentiated SH-SY5Y cell necrosis is induced by 3,4-MDPHP and fentanyl but not 2-Cl-4,5-MDMA. SH-SY5Y cells were exposed to 125 or 250 μM of 3,4-MDPHP (**A**), 2-Cl-4,5-MDMA (**B**), and fentanyl (**C**) for 24 h. Data, expressed as percentage of total cells, represent mean ± SEM from three independent experiments with three replicates for each concentration of the compounds. One-way ANOVA followed by Tukey’s post-hoc, * *p <* 0.05, ** *p <* 0.01 compared to its respective control, # *p <* 0.05 compared to 125 μM of 3,4-MDPHP.

Two-way ANOVA for necrosis displays the main effect of dose (F_(2,18)_ = 8.48, *p* = 0.00250) but not of treatment and dose x treatment interaction. To better evaluate the effect of each treatment, data were analyzed separately. One-way ANOVA revealed a significant concentration-dependent increase in necrotic cells of 3,4-MDPHP-treated cells (250 μM) as compared to the control and cells treated with the lower concentration (*p <* 0.05), while 250 μM of fentanyl induced a significant increase in necrotic cells compared to control-treated cells (*p <* 0.01) (Figure 6A,C). No significant differences in percentage of necrotic cells were observed for all doses tested of 2-Cl-4,5-MDMA (Figure 6B).

#### 2.2.3. 2-Cl-4,5-MDMA and Fentanyl but Not 3,4-MDPHP Altered Bax Expression 

**Figure 7:**
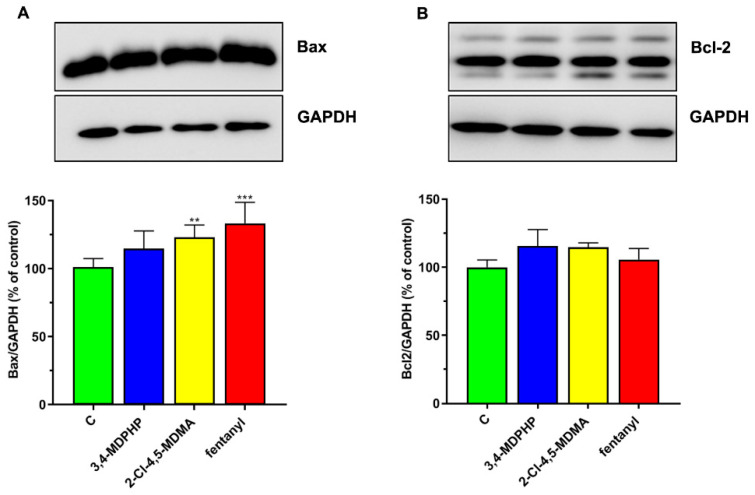
Expression level of Bcl2 and Bax proteins in dopaminergic-differentiated SH-SY5Y cells following treatment with 3,4-MDPHP, 2-Cl-4,5-MDMA, or fentanyl. SH-SY5Y cells were exposed to 250 μM of 3,4-MDPHP, fentanyl, or 2-Cl-4,5-MDMA for 24 h, and levels of Bax (**A**) or Bcl2 (**B**) were measured by Western blot analysis. The protein levels of Bcl2 and Bax in the control and treated cells were quantified and normalized to loading control GAPDH by densitometry, as described in the Material and Methods. Results are presented as mean ± SEM of three independent experiments. One-way ANOVA followed by Tukey’s post-hoc, ** *p <* 0.01, *** *p <* 0.001 compared to control.

Next, to explore the contribution of mitochondrial membrane dysfunction to the drug-induced apoptosis, alterations in expression levels of factors involved in the mitochondrial membrane permeability, such as pro-apoptotic Bax and anti-apoptotic Bcl-2, were examined by Western blot analyses. Treatment of differentiated SH-SY5Y cells with 250 μM of 2-Cl-4,5-MDMA and fentanyl for 24 h resulted in a significant increase with respect to control cells (*p <* 0.001 and *p <* 0.01, respectively) in the expression levels of pro-apoptotic Bax, whereas 3,4-MDPHP induced only a slight but not significant increase. On the other hand, Bcl-2 expression was not modified by any treatments (Figure 7A,B). 

**Figure 8:**
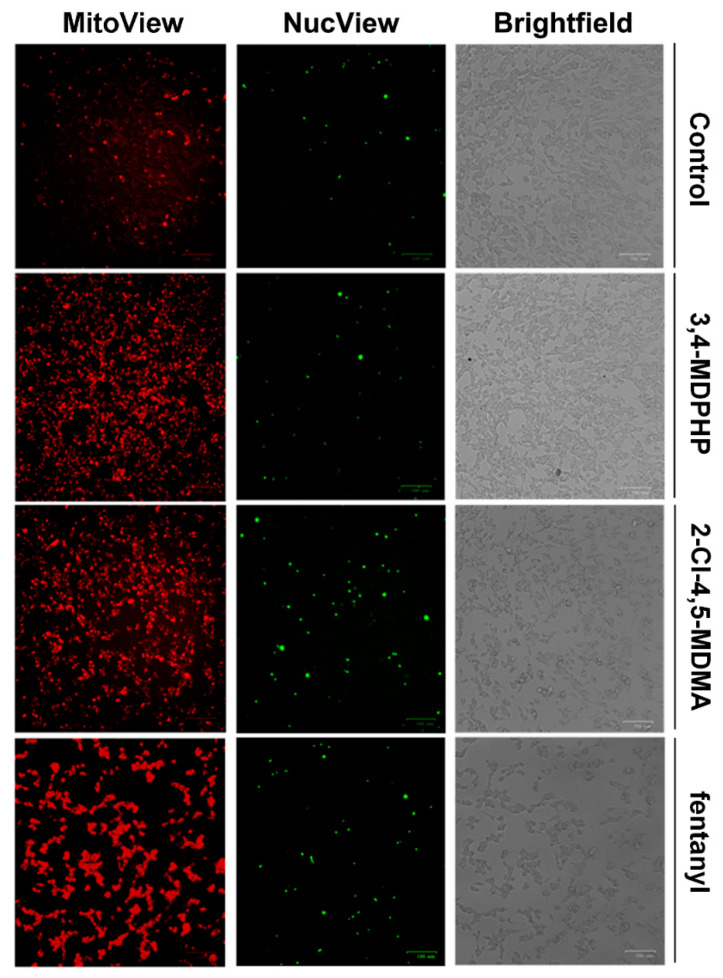
NucView 488 (caspase-3 substrate, green) and MitoView 633 (polarized mitochondria dye, red) staining. After 24 h of treatment with 3,4-MDPHP, fentanyl, or 2-Cl-4,5-MDMA, SH-SY5Y was stained with NucView 488 and MitoView 633 and analyzed by fluorescence microscopy. 2-Cl-4,5-MDMA and fentanyl, but not 3,4-MDPHP, determined an increase in apoptotic cell number (green staining) compared to the control, associated with altered cell morphology (brightfield). All treatments induced mitochondrial hyperpolarization (bar 100 μm).

To further confirm the involvement of the apoptotic process in this treatment-induced neurotoxicity, we profiled apoptotic cells based on the changes in caspase-3 activity. To this aim, NucView^®^ 488, which can detect activated caspase-3, and MitoView™ 633, a membrane potential-sensitive stain for mitochondria, were used. After 24 h of treatment with 250 μM of 2-Cl-4,5-MDMA and fentanyl, the green fluorescence signal (corresponding to the active caspase-3) was higher compared to control cells, indicating an increase in apoptotic cells. In line with previous findings (Figure 5A,B and Table 1) treatment with 3,4-MDPHP had no effect on caspase-3 activity. Red fluorescence signals corresponding to the mitochondrial membrane potential were enhanced in cells treated with all compounds compared to control cells, indicating mitochondrial hyperpolarization that occurs in early phases of apoptosis (Figure 8). Moreover, after 24 h of treatment with 250 μM of fentanyl and 2-Cl-4,5-MDMA, the differentiated SH-SY5Y cells showed morphological alterations, such as shrinkage, loss of processes, round shape, and clumping, indicative of a loss of cell viability and progression toward death (Figure 8 brightfield).

## 3. Discussion

In the present study, we reported for the first time the in vitro neurotoxic effects of two emerging NPS, i.e., 3,4-MDPHP and 2-Cl-4,5-MDMA, belonging to the class of synthetic cathinones and phenethylamines, respectively. Moreover, their cytotoxic effects were compared to that elicited by fentanyl, the reference compound of more recent synthetic opioids that are causing fatal intoxications worldwide. 

Here, we used the dopaminergic-differentiated SH-SY5Y neuroblastoma cell line to investigate the in vitro neuro/cytotoxicity of 3,4-MDPHP, 2-Cl-4,5-MDMA, and fentanyl. This differentiated cell line displays a mature neuron-like phenotype and develops a dopaminergic phenotype, expressing high levels of several dopaminergic markers (i.e., TH, D2, and D3 receptors), thus representing a useful tool to investigate the in vitro neurotoxicity of NPS acting on the dopaminergic system such as synthetic cathinones and phenethylamines [71]. In these cells, we demonstrated that 24 h of treatment with either 3,4-MDPHP, 2-Cl-4,5-MDMA, or fentanyl produced a strong and concentration-dependent inhibition of cell viability, in line with previous studies showing a decreased cell viability following exposure with different cathinones [38]. Specifically, in the dopaminergic-differentiated SH-SY5Y exposed to 3,4-MDPHP, the toxic effects start at 125 and 250 µM (about 16% and 23% reduction in cell viability, respectively) and reach the peak (i.e., 72% decrease in viability vs. control values) at the highest concentration tested (1000 µM). It should be noted that first-generation pyrovalerones (e.g., pyrovalerone, 3,4-MDPV, and 2,3-MDPV) produced only a slight decrease in mitochondrial activity in the examined cell lines, including differentiated SH-SY5Y [72]. Specifically, 24 h of exposure of the banned analog 3,4-MDPV (300 µM) elicits only a modest MTT reduction in SH-SY5Y, although it was active at lower concentrations compared with their reference compound methamphetamine. Additionally, most studies reported that cellular cytotoxicity induced by cathinones is observed only after an incubation time longer than 24 h and with concentrations >1 mM [37,39,40,72]. The sensitivities for inhibition of cell viability might vary according to the different cell lines used, time of exposure, and substituent in synthetic cathinones examined.

Treatment for 24 h with the phenethylamine 2-Cl-4,5-MDMA induced a decrease in MTT, similar to that produced by 3,4-MDPHP, while fentanyl at the same concentrations was more potent than the other two compounds. While high concentrations of synthetic opioids, such as tramadol and tapentadol, caused an increase in toxicity as assessed by MTT assays in the SH-SY5Y cell line [73], no data were available on fentanyl cellular toxicity. Few papers evaluated the cytotoxicity of fentanyl in different cell lines (i.e., human pancreatic cancer cell line, SW1990; human breast carcinoma cell line, MCF-7; microglial cell line, BV-2) and reported cell viability inhibition compared to the control group [74,75,76]. The mitochondrial dysfunction observed following exposure with all the tested compounds did not induce an increase in LDH release, indicating that it was not associated with a loss in membrane integrity. Indeed, the decrease in mitochondrial function often anticipates membrane damage; consequentially, the LDH leakage measurement may be less sensitive compared to the MTT reduction assay [77]. In line with our results, Wojcieszak et al. (2016) showed that MDPV did not trigger LDH leakage in undifferentiated SH-SY5Y cells at concentrations that already exhibited a significant decrease in MTT reduction [72].

Increasing evidence indicate that oxidative stress plays an essential role in the cytotoxic effects of several drugs of abuse (e.g., amphetamines and cathinones) inducing the formation of highly reactive species [78,79]. In vitro studies have shown cytotoxic effects of all cathinone derivatives in several cell lines, reporting increased ROS/RNS production, and/or the depletion of reduced glutathione, as well as increased oxidized glutathione levels [38]. The cytotoxicity of 3,4-MDPV, a progenitor of α-pyrrolidinophenones and an analog of 3,4-MDPHP, has been reported in neuronal and hepatic cell lines, along with their mechanisms of toxicity, which are the disruption of mitochondrial function, and the oxidative stress (generation of reactive oxygen and nitrogen species, depletion of reduced glutathione). Regarding the phenethylamines, a recent study showed the ability of some of these compounds (i.e, 2C-H, 2C-I, 2C-B, and 25B-NBOMe) to induce an ROS increase in the human lymphoblastoid TK6 cells 1 h after treatment, while MDMA failed in increasing ROS levels [44]. Accordingly, Valente and collaborators showed that MDMA can increase ROS levels in a primary rat hepatocytes cell line [40] but not in undifferentiated and differentiated human SH-SY5Y cells [41]. Our findings demonstrated that, in line with studies showing an ROS increase in dopaminergic-differentiated SH-SY5Y cells, 3,4-MDPHP and 2-Cl-4,5 MDMA increase ROS levels to a similar extent, while a stronger production of ROS is observed after exposure to the highest concentration of fentanyl. Currently, due to a lack of information in the literature, it is not possible to compare our data concerning increases in fentanyl-induced cytotoxicity and ROS levels. Following the oxidative damage, mitochondria may go through profound changes, including outer membrane permeabilization and subsequent translocation of the pro-apoptotic factor Bax. This factor leads to the release of cytochrome C that promotes the activation of caspases, which, in turn, manage apoptosis through the cleavage of numerous proteins, leading to the engulfment of the dying cells [80]. Apoptosis is a genetically encoded programmed cell death, while necrosis is typically not associated with activation of caspases and is thought to mediate cell death in response to damage or a pathology [81]. The present data reveal that our compounds provoke cell death through different mechanisms, both apoptosis and necrosis, with 2-Cl-4,5-MDMA mainly activating the apoptotic process, 3,4-MDPHP mainly by necrosis, and fentanyl inducing both mechanisms. Specifically, 2-Cl-4,5-MDMA and fentanyl treatment induce early apoptosis features (i.e., stained only with annexin V-FITC) in a high number of cells, while cells treated with 3,4-MDPHP showed necrosis features (i.e., stained with propidium). 

Furthermore, the observed increase in expression levels of pro-apoptotic Bax and caspase 3 activity following 2-Cl-4,5-MDMA, but not 3,4-MDPHP treatment, confirm the different modes of cell death, i.e., apoptosis and necrosis for 2-Cl-4,5-MDMA and 3,4-MDPHP, respectively. In line with these results, apoptotic and necrotic phenomena were previously described following abuse of piperazines [82], mephedrone [83], and related cathinones [84]. The treatment with fentanyl induces a high number of either annexin V-FITC and/or PI-stained cells, and increased levels of Bax and caspase 3 activity, supporting a double mechanism of cell death. It should be noted that the concentrations of drugs used in our in vitro study are higher than those found in urine or blood of drug abusers [85,86,87]; however, our data are consistent with several studies reporting in vitro cytotoxic effects (i.e., reduced viability at concentrations >1–2 mM) after exposure to several drugs of abuse, including cathinones, amphetamine-like stimulants, phenethylamines, and synthetic cannabinoids [41,88,89,90,91,92]. It should also be considered that, due to their high lipophilicity, fentanyl and cathinone derivatives possess a high volume of distribution, reaching significantly greater concentrations in lipophilic tissues, such as the central nervous system [93], and consequently, their blood concentrations might not directly reflect their organ/tissue levels. Moreover, there is a lag time between drug intake and blood samples collection, and that may result in lower concentrations being identified due to metabolic processes and the antemortem/postmortem redistribution of the lipid-soluble compounds. 

In conclusion, this collection of data provides evidence that the emerging NPS, 2-Cl-4,5-MDMA, and 3,4-MDPHP induce in vitro neurotoxicity associated with oxidative stress (increase in ROS) but trigger cell death through different mechanisms. Indeed, while 2-Cl-4,5-MDMA activates apoptotic processes, 3,4-MDPHP causes cell death mainly by necrosis. Fentanyl instead was able to induce both mechanisms displaying higher potency. Furthermore, this series of in vitro tests might serve as a quick and efficient way to screen new compounds to have useful information about their neurotoxicity, and to direct further in silico and in vivo studies for a full pharmacological and toxicological characterization. 

## 4. Materials and Methods

### 4.1. Drugs 

1-(6-chlorobenzo[d][1,3] dioxol-5-yl)-*N*-methylpropan-2-amine, monohydrochloride (2-Cl-4,5-MDMA); 1-(1,3-benzodioxol-5-yl)-2-(1-pyrrolidinyl)-1hexanone, monohydrochloride [3,4-methylenedioxy-α-pyrrolidinohexanophenone] (3,4-MDPHP); and N-phenyl-*N*-[1-(2-phenylethyl)-4-piperidinyl]-propanamide, monohydrochloride (fentanyl) were purchased from Cayman Chemical (Cayman Chemical, Ann Arbor, Michigan USA). Stock solutions of each compound were prepared in 100% DMSO. These stock solutions were sequentially diluted in DMEM before cell exposure to obtain a final DMSO concentration not exceeding 1% (vol/vol), which our preliminary experiments showed to be nontoxic (data not shown).

### 4.2. Reagents 

Retinoic acid (RA), dimethyl sulfoxide (DMSO), (4,5-dimethylthiazol-2-yl)-2,5-diphenyltetrazolium bromide (MTT), and 2′,7′-dichlorofluorescin diacetate probe (H2-DCF-DA) were purchased from Merck Life Science (Milan, Italy). The Lactate dehydrogenase (LDH) Assay Kit was obtained from Abcam (Cambridge, UK), while the dead Cell Apoptosis Kit with Annexin V and propidium iodide (PI) and the Pierce™ BCA Protein Assay Kit from Thermo Fisher Scientific (Waltham, MA USA), NucView^®^ 488, and MitoView™ 633 Apoptosis Assay Kit were obtained from Biotium (Fremont, CA, USA). 

### 4.3. Cell Culture and RA-Differentiation 

Human neuroblastoma cell line SH-SY5Y was kindly provided by Dr. Miceli (University of Naples Federico II, Naples, Italy). Cells were cultured in high glucose DMEM supplemented with 10% heat-inactivated fetal bovine serum, 100 units/mL of penicillin, and 100 µg/mL of streptomycin (all from Gibco, Life sciences; Thermo Fisher Scientific, Waltham, MA USA), and maintained at 37 °C in a humidified atmosphere of 5% CO2 and 95% air. The medium was changed twice a week and cells were split at about 80% confluence. To induce dopaminergic differentiation, cells were treated with 10 μM of RA every 48 h for 7 days. SH-SY5Y cells were used for all the experiments between the 28th and 35th passages, to avoid phenotypic changes.

### 4.4. Cytotoxicity Studies 

To evaluate the cytotoxic properties of 3,4 MDPHP, 2-Cl-4,5 MDMA, and fentanyl, MTT and LDH assays were performed.

#### 4.4.1. MTT Assay 

Differentiated SH-SY5Y cells were plated at the density of 1.5 × 10^4^ cells/well in 96-well plates and incubated for 24 h with different concentrations (15–1000 μM) of compounds. After incubation, 50 μL of MTT reagent (1 mg/mL in DMEM) was added and cells were incubated for 4 h at 37 °C. The resulting formazan crystals were dissolved in 100 μL of DMSO and quantified by spectrophotometry at 540 nm using a TECAN microplate reader (Infinite 200, Tecan, Salzburg, Austria). Viability data were reported in percentage of control (vehicle cells) for each compound.

#### 4.4.2. LDH Assay 

Differentiated SH-SY5Y cells were plated at a density of 1.5 × 10^4^ cells/well in 96-well plates and incubated for 24 h with compounds (125 and 250 μM). After 24 h of treatment, the activity of the cytoplasmic enzyme LDH released into the culture medium was measured using an LDH-cytotoxicity assay kit according to the manufacturer’s indications. The results are expressed as percentage of LDH released by control cells.

### 4.5. Determination of Intracellular Reactive Oxygen (ROS) Production

For ROS determination, differentiated SH-SY5Y cells were seeded at a density of 1.5 × 10^4^ cells/well in 96-well plates. Cells were then treated for 24 h with compounds (125 and 250 μM) or culture medium containing 1% of DMSO as a negative control. After incubation, cells were washed with PBS and incubated for 30 min with 10 μM of H_2_-DCF-DA. As a positive control, hydrogen peroxide (H_2_O_2_, 100 μM) was used. H_2_-DCF-DA was replaced with PBS and ROS levels were measured by using a Tecan micro plate reader at a controlled temperature of 37 °C. The reading was performed using an excitation of 490 nm and an emission of 520 nm. The results are expressed as a fold-increase with respect to the control from three independent experiments with each concentration tested in five replicates within each experiment.

### 4.6. Cell Death Assay 

To investigate the cell death induced by the treatment of compounds, a flow cytometric analysis was performed using the Annexin V-FITC/PI Apoptosis detection kit. Differentiated SH-SY5Y cells were plated in 6-well plates at the density of 3 × 10^5^ cell/well and were then treated with 125 and 250 μM of compounds for 24 h. After trypsinization, cells were washed once with PBS and re-suspended in 100 μL of Annexin binding buffer plus 5 μL of Annexin V fluorescein isothiocyanate and 1 μL of PI. After incubation in the dark for 15 min at room temperature, stained cells were analyzed by flow cytometry, measuring the fluorescence emission at 530 and 620 nm using a 488 nm excitation laser (MoFloAstrios EQ, Beckman Coulter) (Brea, CA, USA). Cell apoptosis was analyzed using Software Summit Version 6.3.1.1, Beckman Coulter. 

### 4.7. Bcl2 and Bax-2 Detection in SH-SY5Y-Differentiated Cells 

After 24 h of treatment with 250 μM of 3,4-MDPHP, 2-Cl-4,5 MDMA, or fentanyl, dopaminergic-differentiated SH-SY5Y cells were lysed in 2% sodium dodecyl sulphate (SDS). The Pierce BCA assay was used to quantify the protein concentration of each sample. Equal amounts of protein (15 ug) were denatured at 100 °C for 3 min in Laemmli loading buffer, separated on 12% polyacrylamide by sodium dodecyl sulphate-polyacrylamide gel electrophoresis (SDS/PAGE) and transferred to polyvinylidene fluoride (PVDF) membranes (Hybond-P, Amersham, Marlborough, MA, USA). Membranes were blocked with 5% nonfat dry milk for 1 h at RT and then incubated with the primary rabbit monoclonal anti Bax antibody (1:1000; #32503, Abcam, Cambridge, UK ) or rabbit polyclonal anti Bcl-2 (1:500; #196495 Abcam, Cambridge, UK) overnight at 4 °C; blots were also probed for mouse monoclonal anti-GAPDH (1:1000; Mab 374 Merck Life Science, Milan, Italy) to evaluate equal loading. The membranes were then washed and further incubated with horseradish-peroxidase-conjugated antirabbit IgG (1:10,000; #111-035-003 Jackson ImmunoResearch, Ely, UK) or anti mouse IgG (1:10,000; #115-035-003 Jackson ImmunoResearch, Ely, UK) for 1 h at RT. After washing, protein bands were detected with a chemiluminescent substrate (LiteAblot TURBO, Euroclone, Italy) and visualized by ImageQuant LAS-4000 (GE Healthcare, Little Chalfont, UK). Band intensities were quantified using Image Studio (Li-Cor, Lincoln, USA) and normalized against GAPDH. Results were expressed as percentage with respect to the control (cells treated with the vehicle).

### 4.8. Apoptosis and Mitochondrial Activity Assay

SH-SY5Y cells were seeded onto 24-well plates at 3 × 10^4^ cells per well and differentiated for 7 days as described above. Cells were exposed for 24 h to 250 μM of 3,4 MDPHP, 2-Cl-4,5 MDMA, fentanyl, or DMSO 1% (vehicle control). After 24 h, NucView™ 488 (caspase 3/7 substrate) and MitoView™ 633 (mitochondrial dye) probe solutions were added, following the manufacturer’s instructions. The probes were incubated for 30 min and then cells were visualized using ZOE Fluorescent Cell Imager (Bio-Rad) at a magnification of 20X. Instrument gain and offset values were adjusted using the negative control and remained constant for all subsequent experiments.

### 4.9. Statistical Analysis

The results are the mean of at least three independent experiments, performed at least in quadruplicate. Data are expressed as mean ± SEM and differences were statistically significant at *p* < 0.05. Normality tests for data were carried out using Shapiro–Wilk’s test. If data were found to be normally distributed, the effect of treatment was analyzed using one-way ANOVA for MTT, LDH assay, and Western blot analysis. ROS determination and apoptosis data were analyzed by two-way ANOVA (dose x treatment) followed by Tukey’s post-hoc test. Statistical analysis was performed with Statistica 10.0 (StatSoft, Tulsa, OK, USA) or GraphPad Prism 8 software (GraphPad Prism, RRID:SCR_002798) (San Diego, CA, USA)

## Figures and Tables

**Table 1:** Percentage of early and late apoptotic and necrotic SH-SY5Y cells following treatment with 3,4MDPHP, 2-Cl-4,5-MDMA, and fentanyl, measured by flow cytometry.

	Control	Fentanyl		2-Cl-4,5 MDMA		3,4-MDPHP	
		125 μM	250 μM	125 μM	250 μM	125 μM	250 μM
Live	87.88 ± 2.13	81.03 ± 2.36	73.50 ± 5.02	64.34 ± 6.74	55.81 ± 1.79	82.44 ± 2.84	78.94 ± 3.99
Early apoptotic	1.93 ± 1.11	6.83 ± 0.82	10.53 ± 1.45	21.21 ± 2.36	29.26 ± 4.05	4.24 ± 1.89	3.00 ± 1.92
Late apoptotic	1.30 ± 0.85	0.64 ± 0.26	1.43 ± 0.46	2.96 ± 1.32	5.07 ± 2.26	1.81 ± 0.67	1.46 ± 0.82
Necrotic	8.89 ± 1.28	11.50 ± 0.19	14.54 ± 2.69	11.49 ± 4.48	9.85 ± 3.47	11.51 ± 2.11	16.60 ± 2.65

Data represent the mean ± SEM from three independent experiments with three replicates for each concentration of the compounds.
